# Multidimensional crisis in the conservation of Amu Darya false shovelnose sturgeons

**DOI:** 10.1038/s44185-026-00141-w

**Published:** 2026-06-01

**Authors:** Akbarjon Rozimov, Xinxin Li, Bakhtiyor Sheraliev, Alexey Chernyak, Zakir Rajabov, Jobir Sobirov, Laziz Turaev, Sirojiddin Namozov, Chongnv Wang, Bakhtiyor Kholmatov, Zuogang Peng, Arne Ludwig, Baocheng Guo

**Affiliations:** 1 State Key Laboratory of Animal Biodiversity Conservation and Integrated Pest Management, Institute of Zoology, Chinese Academy of Sciences, Beijing, China; 2 University of Chinese Academy of Sciences, Beijing, China; 3 Institute of Zoology, Academy of Sciences of the Republic of Uzbekistan, Tashkent, Uzbekistan; 4 IUCN Sturgeon Specialist Group, Gland, Switzerland; 5 Department of Zoology and General Biology, Faculty of Life Sciences, Fergana State University, Fergana, Uzbekistan; 6 Eurasian Regional Association of Zoos and Aquariums, Moscow, Russia; 7 Khorezm Mamun Academy, Academy of Sciences of the Republic of Uzbekistan, Khiva, Uzbekistan; 8 School of Life Sciences, Southwest University, Chongqing, China; 9 Department of Evolutionary Genetics, Leibniz Institute for Zoo and Wildlife Research, Berlin, Germany

**Keywords:** Ecology, Ecology, Evolution, Genetics, Zoology

## Abstract

A robust reassessment of extinction risk is crucial for developing evidence-based conservation strategies and preventing irreversible biodiversity loss. Sturgeons are among the most threatened freshwater fishes worldwide, yet extinction risk evaluations for the critically endangered Amu Darya false shovelnose sturgeons (*Pseudoscaphirhynchus kaufmanni* and *P. hermanni*) still largely depend on legacy information that does not reflect recent human disturbances. To re-evaluate the extinction risk of these sturgeons, we combined updated occurrence records, traditional morphometric data, mitochondrial genetic markers, and ethnobiological surveys from field expeditions, market inspections, and community interviews conducted during 2019–2024. Our results showed dramatic contraction in distribution range and an almost complete loss of population structure. The long-snouted form of *P. hermanni* is likely already extinct, and the short-snouted form now persists only in fragmented habitats in the middle and lower Amu Darya. Morphological comparisons revealed a marked decrease in body length and weight, indicating size-selective removal linked to illegal poaching. Genetic analyses also revealed declining diversity and signals of demographic bottlenecks, consistent with severely reduced adaptive potential. Taken together, our results indicate an urgent need for coordinated transboundary conservation to avert the imminent extinction of the two critically endangered species.

## Introduction

Although freshwater covers only 0.8% of the Earth’s surface, it remarkably sustains approximately 43% of all known fish species^1–3^. That imbalance, a small area with immense biodiversity, is now being rapidly altered by escalating human pressures. This places freshwater biota among the most threatened groups worldwide. The IUCN Red List of Threatened Species shows that nearly one-quarter of freshwater species are at risk of extinction^4,5^. The acceleration of worldwide biodiversity losses makes it critical to regularly reassess species’ status with robust evidence, which could inform conservation priorities and prevent irreversible declines. Sturgeons and paddlefish (Acipenseriformes), are particularly vulnerable due to their life-history traits, such as slow growth, large body size, late maturation, and extreme longevity, which make them poorly buffered against human disturbance. Nowadays, 63% (17 of 27) of acipenseriform species are listed as Critically Endangered or Extinct on the IUCN Red List^6^, making them one of the most threatened groups of all fish species.

**Fig. 1:**
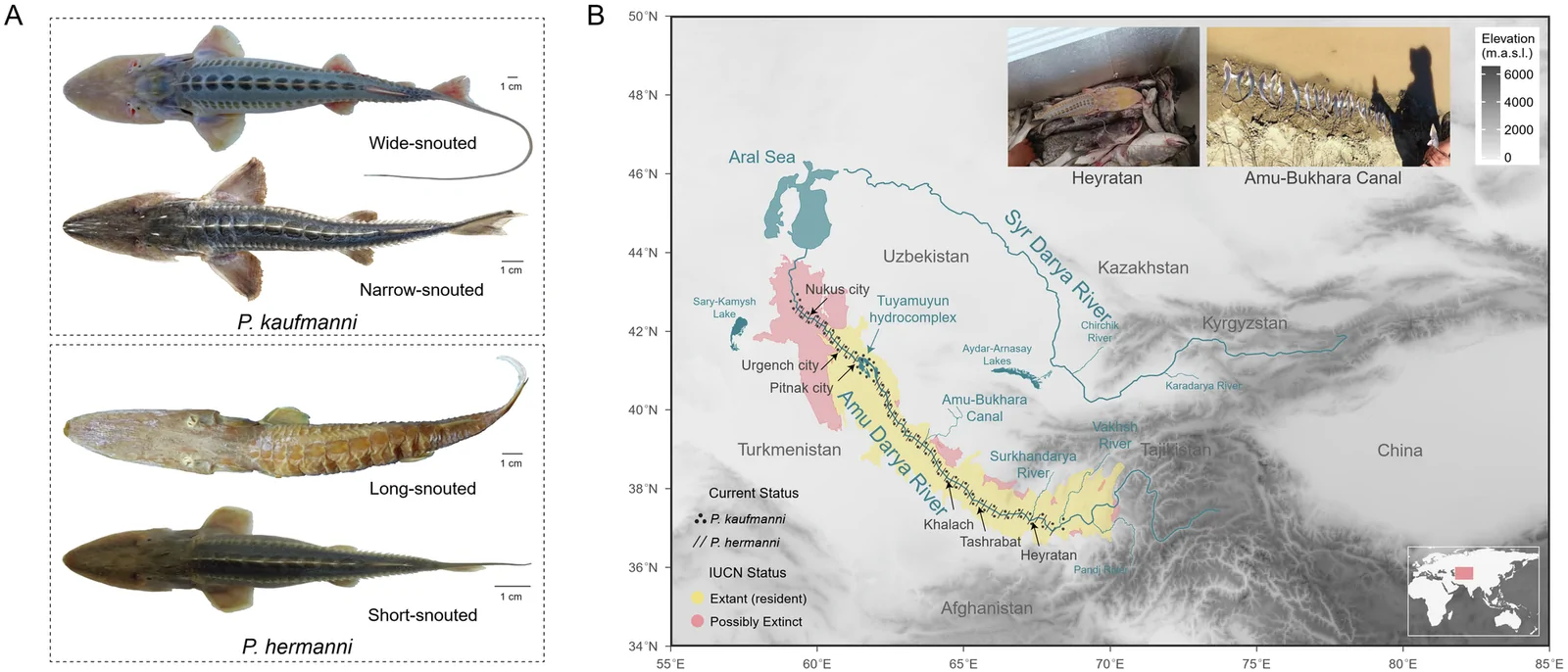
Morphotypes and spatiotemporal shifts in the distribution of Amu Darya false shovelnose sturgeon. **A** Morphotypes of the big Amu Darya false shovelnose sturgeon (*P. kaufmanni*) and the small Amu Darya false shovelnose sturgeon (*P. hermanni*). The caudal tail filament of the narrow-snouted *P. kaufmanni* was broken due to injury. **B** Comparison of current and historical distribution ranges for Amu Darya false shovelnose sturgeons.

The Aral Sea basin, once a biogeographic stronghold for sturgeon diversity, initially harbored the anadromous Aral ship sturgeon (*Acipenser nudiventris*) and three endemic potamodromous false shovelnose sturgeons, namely the Syr Darya false shovelnose sturgeon (*Pseudoscaphirhynchus fedtschenkoi*), big Amu Darya false shovelnose sturgeon (*P. kaufmanni*), and small Amu Darya false shovelnose sturgeon (*P. hermanni*) (Fig. 1A)^7–11^. However, this once-thriving species diversity has disappeared due to the ecological collapse of the Aral Sea^12–15^. This ecological collapse was primarily caused by river engineering in the Soviet era, exemplified by the Takhiatash and Tuyamuyun dams, and by massive water withdrawals to support industrial agriculture. These changes blocked migration corridors, disrupted spawning habitats, and fundamentally altered the river’s flow regime. These anthropogenic disruptions precipitated the presumed extinction of *P. fedtschenkoi*^16^, the disappearance of the Aral Sea subpopulation of *A. nudiventris*^13,17^, and the critical decline of both *P. hermanni*^13,18^ and *P. kaufmanni*^10,19,20^. In response, the IUCN Red List listed *P. kaufmanni* as Endangered and *P. hermanni* as Critically Endangered in 1996. Both species were listed as Critically Endangered in 2010 and again in 2022. However, all these evaluations heavily rely on outdated evidence, thereby hindering effective conservation planning. With human activity pressures intensifying and climate risks escalating, an updated reassessment is necessary to guide interventions and prevent the loss of these evolutionarily distinctive lineages.

We reassessed the status of Amu Darya false shovelnose sturgeons by combining field surveys with morphometric comparisons, analyses of mitochondrial genetic diversity, and sociological research. To map the current distribution of Amu Darya false shovelnose sturgeons, especially that of the emblematic *P. hermanni*, we combined our field observations with verified public news reports. Comparisons with historical specimens revealed that contemporary fish are significantly smaller, and analyses of *cyt b* and control region sequences indicated reduced genetic diversity consistent with recent demographic bottlenecks. The observed loss of critical spawning aggregations and pronounced morphological shifts suggest that illegal poaching is a key driver of the population collapse. The combined evidence of phenotypic degradation, genetic erosion, and unsustainable exploitation highlights the urgent need for coordinated conservation interventions across regional and international scales to mitigate ongoing threats to these critically endangered sturgeons.

## Methods

### Data acquisition and sampling

To assess the conservation status of Amu Darya false shovelnose sturgeons, we carried out field expeditions from 2019 to 2024 along a 202-km reach of the Amu Darya River in Uzbekistan, from Pitnak (Khorezm; 41°14′23.4”N, 61°18′54.6”E) to Nukus (Karakalpakstan; 42°32′01.4”N, 59°35′24.9”E), following the survey protocols of Salnikov, et al.^21^. Our field and bycatch surveys yielded a total of 13 short-snouted *P. hermanni* individuals from 2020 to 2024 (Table S1). Of these, seven were live-captured and immediately released, while 6 dead individuals were used for morphological and genetic analyses. For genetic analysis, pectoral fin clips were collected from 31 Amu Darya false shovelnose sturgeons and preserved in 96% ethanol (Table S1). Supplementary records of Amu Darya false shovelnose sturgeons were obtained from the Global Biodiversity Information Facility.

To investigate the scale of the wild-capture trade and describe market dynamics, we conducted undercover market surveys for *P*. *kaufmanni* in Khorezm from 2021 to 2023. We also interviewed fishers and traders in major districts of Khorezm (Khazarasp, Khanka, Urgench, Yangibozor, and Gurlan) and Karakalpakstan (Tortkol, Beruni, and Nukus). Over the three years, we recorded 170 illegally traded *P. kaufmanni*, in addition to seven individuals encountered during field surveys (Table S1). To examine sociocultural drivers of poaching, a structured social perception survey was administered in 2021 via the Facebook group “Khorezm and Khorezmian (https://www.facebook.com/share/p/1GKgJoFKDT/?mibextid=wwXIfr)”. The survey quantified the prevalence of the widespread misconception that *P. kaufmanni* possesses medicinal properties for treating infertility. This unfounded belief has become a major driver of illegal fishing and trade.

All Amu Darya false shovelnose sturgeon monitoring and sampling procedures were carried out following the requirements of current Uzbek environmental legislation and the Law of the Republic of Uzbekistan “On the protection and use of wildlife” (No. LRU-408 19.09.2016; https://lex.uz/ru/docs/-3029502).

### Comparative morphometry analysis

A total of 22 *P. kaufmanni* individuals (19 wide-snouted and 3 narrow-snouted) and 6 short-snouted *P. hermanni* individuals were included in the morphometric and length-weight relationship (LWR) analyses. Historical morphometric data were obtained from Salnikov, et al.^21^. Major morphometric traits were measured on the left side of each specimen following the methodology of Tleuov and Sagitov^10^. Temporal changes in body morphometry from 1996 to 2019–2024 were analyzed in R v4.3.2. Before parametric analyses, all measurements were log-transformed, and the normality of the transformed data was assessed with the Shapiro–Wilk test. The summary statistics of the log-transformed morphometric traits and the results of the Shapiro–Wilk normality tests are provided in Table S2. Variance homogeneity was examined using both a pairwise F-test (var.test, R base *stats*) and Levene’s test (leveneTest, *car* package). We then applied two-way ANOVAs to *P. kaufmanni* to evaluate the effects of morphotype, time period, and their interaction on the log-transformed traits. The LWR was calculated using the following equation:

$$W=a{{\rm{TL}}}^{b}$$

and logarithmically transformed^22^ into:

$$\log \left(W\right)=\log \left(a\right)+b* \log ({\rm{TL}})$$

*W*, TL, *a*, and *b* are the body weight, total body length, the intercept, and the slope, respectively. The 95% confidence intervals for *a* and *b*, and the coefficient of determination (R^2^) were calculated using the equations of Sparre^23^. Specifically, TL was measured from the snout tip to the end of the upper lobe of the caudal fin, excluding the caudal filament, which was damaged in one *individual of P. kaufmanni*. Measurements were recorded to the nearest 0.1 cm for TL (without the caudal filament) and to the nearest 0.01 g for *W*. All statistical analyses were performed in R v4.3.2.

### Sequencing, mitochondrial genome assembly, and genetic diversity estimation

Whole-genome resequencing was performed for 31 Amu Darya false shovelnose sturgeons, including 26 *P. kaufmanni* individuals and 5 *P. hermanni* individuals. All experimental procedures were conducted by Biomarker Technologies (Beijing, China). Approximately 1 Gb of raw resequencing data was generated for each individual. Mitochondrial genomes were assembled using GetOrganelle v1.7.7.0^24^ with default parameters. Publicly available *cyt b* and CR sequences of *P. kaufmanni* and *P. hermanni* were retrieved from Dillman, et al.^25^. Sequences were concatenated with SeqKit v2.8.2^26^, aligned with MAFFT v7.525^27^, and trimmed with TrimAl v1.5.0^28^. Genetic diversity parameters, including the number of polymorphic sites, number of haplotypes, Hd, and π, were estimated using DnaSP v6.12.03^29^, and statistical significance was assessed through 1000 permutations. Temporal changes in Hd and π between 1997–2005 and 2020–2024 were evaluated using two-tailed Z-tests, with the test statistic calculated as Z = (x₁ − x₂) / √(SE₁² + SE₂²). All analyses were performed in R v4.3.2 using base R functions.

## Results

### Current distribution and range contraction of Amu Darya false shovelnose sturgeons

The current distribution of Amu Darya false shovelnose sturgeons (Fig. 1B) was reconstructed by combining our field data from 2019 to 2024 with published historical surveys (1989–2020) (Salnikov et al.^21^; Zholdasova^13^; Grischenko et al.^30^; Sal’nikov et al. 2013; Artaev et al.^31^), and official government sources (https://t.me/ecogovuz/2862). Integrated field observations and bycatch analyses confirmed that *P. kaufmanni* currently occupies nearly the entire course of the Amu Darya River (Fig. 1B and Table S1). Photographic documentation from Heyratan, Afghanistan (Fig. 1B), verified the presence of remnant populations in the headwater thalweg zone of the upper basin. In addition, a report from the Bukhara State Ecology Committee recorded the capture of 24 narrow-snouted *P. kaufmanni* individuals in the Amu-Bukhara Canal, a diversion channel linked to the middle Amu Darya (Fig. 1B, https://t.me/ecogovuz/2862). In comparison, the lower Amu Darya, particularly the Pitnak-Urgench stretch in the Khorezm region, remains a relative stronghold for the species, yielding peak net captures of 12 individuals per night in 2021. In contrast, *P. hermanni* is now confined mainly to the middle and lower reaches of the Amu Darya (Fig. 1B). Although seven individuals were captured in the lower Vakhsh River, a tributary of the upper Amu Darya, during 1995–1997^30^, neither the present surveys (2019–2024) nor prior efforts by N. Mirzoev (2005–2020) detected any individuals in this region of Tajikistan (Fig. 1B)^31^. Historical observations of 25 individuals between Tashrabat and Khalach in Turkmenistan (Fig. 1B) during 1996–2002^32^ highlighted the former abundance of *P. hermanni* in the middle Amu Darya. Notably, after a 20-year absence, the short-snouted morphotype reappeared in the lower Amu Darya^33^, with 13 individuals recorded between Pitnak and Nukus from 2020 to 2024 (Fig. 1B). In contrast, the long-snouted morphotype has not been confirmed since a single verified record in 1996^21^, indicating a probable extirpation.

### Temporal morphological change and variance contraction

**Fig. 2:**
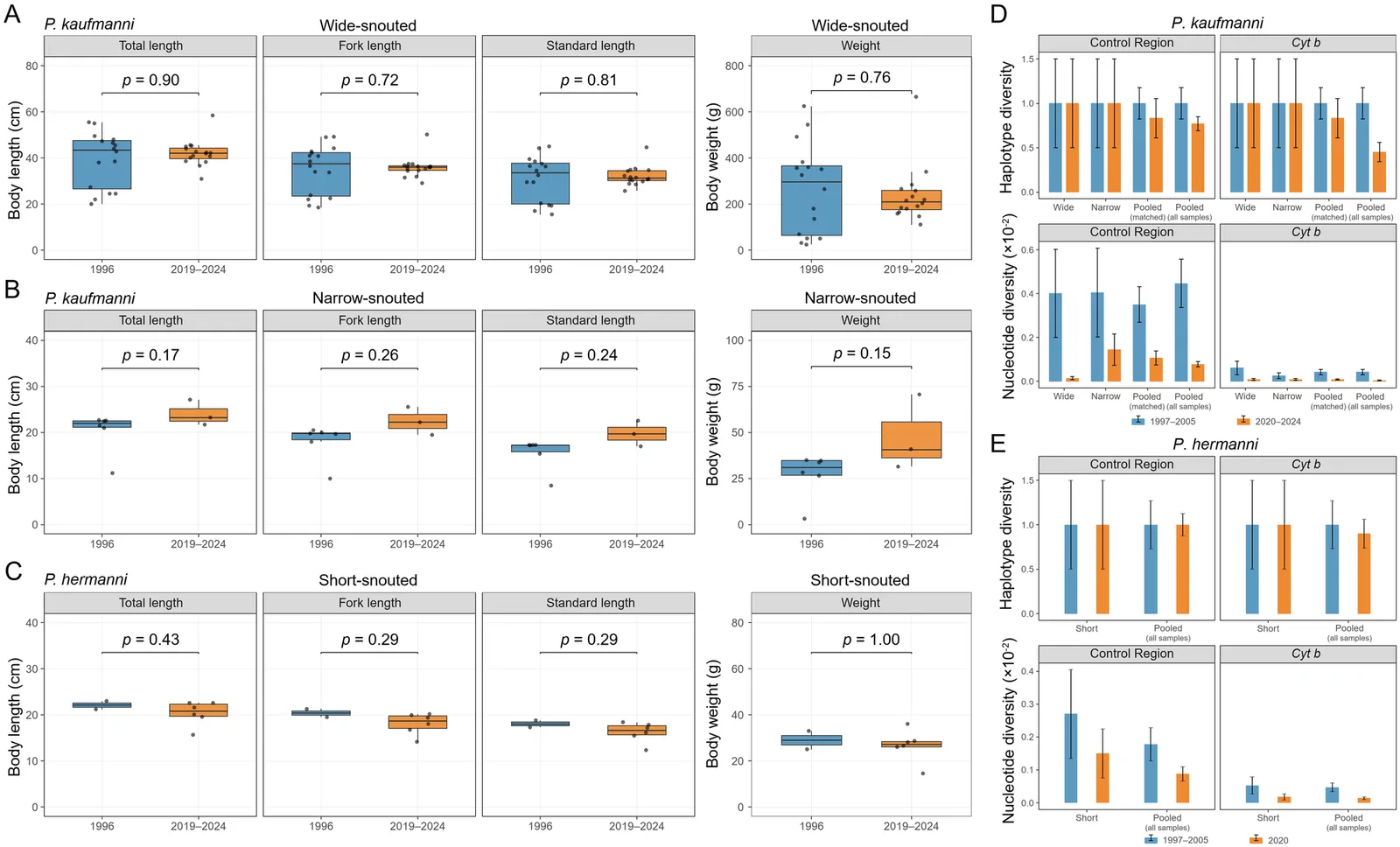
Temporal shifts in morphometric features, body weight, and genetic diversity of Amu Darya false shovelnose sturgeon. **A–C** Morphometric and body weight comparisons among wide-snouted, narrow-snouted, and short-snouted forms of specimens sampled in different periods. **D**, **E** Temporal changes in genetic diversity for *P. kaufmanni* and *P. hermanni* based on *cyt b* and control region (CR), respectively. Wide, wide-snouted; Narrow, narrow-snouted; Short, short-snouted; Pooled (matched), wide- and narrow-snouted samples pooled at a matched sampling level in each period; Pooled (all samples), wide- and narrow-snouted samples pooled using all available sequences in each period for *P. kaufmanni*, and short-snouted samples and long-snouted records from a previous study for *P. hermanni*. Sample sizes (n): wide-snouted *P. kaufmanni* (1996, *n* = 16; 2019–2024, *n* = 16); narrow-snouted *P. kaufmanni* (1996, *n* = 6; 2019–2024, *n* = 3); short-snouted *P. hermanni* (1996, *n* = 2; 2019–2024, *n* = 6).

Comparative analysis of historical and contemporary *Pseudoscaphirhynchus* specimens (Fig. 2A–C and Table S3) revealed morphotype-specific morphological changes. In wide-snouted *P. kaufmanni*, total length (TL) showed a non-significant decline from 1996 to 2019–2024 (median 43.40 cm vs. 42.05 cm; Wilcoxon rank-sum test, *p* = 0.90; Fig. 2A and Table S3), accompanied by a marked contraction in size dispersion, with the interquartile range (IQR) narrowing from 20.88 cm to 4.53 cm, reflecting a substantially more homogeneous size structure in contemporary populations. Fork length (FL) and standard length (SL) (Fig. 2A) exhibited similar compression. This phenotypic homogenization is consistent with two non-mutually exclusive mechanisms: selective removal of larger individuals through size-biased poaching, as evidenced by an increase in minimum size thresholds from 20.00 to 30.92 cm, and demographic collapse linked to habitat fragmentation, which likely reduced effective population size and depleted genetic variation. Although the body weight difference was not statistically significant, the pattern was more pronounced than for length, with the median body mass dropping from 295.50 g to 209.05 g (Wilcoxon rank-sum, *p* = 0.76). In other words, the fish appear to be losing weight faster than they are growing shorter. This kind of thin-out pattern is consistent with long-term, low-level stress in the river—less food to grow on, or chronic pollutant exposure that interferes with endocrine regulation—both of which can divert energy away from growth and into basic maintenance^34,35^. In contrast, narrow-snouted *P. kaufmanni* exhibited increases across all metrics: TL increased by 5.68% (22.00 cm to 23.25 cm), FL by 12.61% (19.75 cm to 22.24 cm), SL by 14.09% (17.25 cm to 19.68 cm), and weight by 31.35% (31.00 g to 40.72 g), although none of these differences reached statistical significance (Wilcoxon rank-sum, all *p* ≥ 0.15) (Fig. 2B and Table S3). These patterns coincided with a drop in the number of observed individuals (from 6 to 3). That reduction is consistent with density-dependent growth release—when fewer fish remain, competition eases, and the survivors can assimilate more resources. Notably, we did not encounter any juveniles (the minimum TL increased from 11.2 cm to 21.72 cm), pointing to poor recruitment. This likely reflects, at least in part, altered flow regimes associated with dam construction, which can disrupt the hydrological cues needed to trigger spawning. For short-snouted *P. hermanni*, individuals sampled in 2019–2024 were marginally smaller than those recorded in 1996 across all length measures: TL (20.83 vs. 22.10 cm), FL (18.71 vs. 20.40 cm), and SL (16.65 vs. 18.05 cm), although none of these differences was significant (Wilcoxon rank-sum test, *p* ≥ 0.29; Fig. 2C). We also observed a slight reduction in body weight (27.24 g vs. 29.00 g) (Wilcoxon rank-sum test, *p* = 1.00). The narrow range in both body weight and body length may reflect stabilizing selection imposed by chronic anthropogenic pressures, such as long-term pollution exposure, or genetic erosion due to hybrid breakdown from introgression with non-native strains, potentially constraining phenotypic plasticity in body size.

To assess these patterns within a more integrated framework, we analyzed log-transformed traits in *P. kaufmanni* using a two-way ANOVA, with morphotype, period, and their interaction as explanatory variables (Table 1). Among the four variables examined, morphotype displayed a highly significant influence (all *p* < 0.001), while the period did not show significance for TL, FL, SL, or body weight (*p* = 0.124, 0.187, 0.153, and 0.222, respectively). Additionally, the interaction between morphotype and period was also non-significant across all measures (*p* = 0.682, 0.399, 0.393, and 0.438, respectively). Thus, after accounting for morphotype, we found no significant temporal shift in mean body size within *P. kaufmanni*.

**Table 1:** Two-way ANOVA of log-transformed morphometric traits in *Pseudoscaphirhynchus kaufmanni* (morphotype × period)

Trait	Source of variation	df	SS	MS	F	η²	*P*-value
Total length	morphotype	1	2.795	2.795	42.458	0.517	0.000
	period	1	0.163	0.163	2.476	0.030	0.124
	morphotype × period	1	0.011	0.011	0.171	0.002	0.682
	residuals	37	2.436	0.066	NA	0.451	NA
Fork length	morphotype	1	2.494	2.494	39.858	0.502	0.000
	period	1	0.113	0.113	1.809	0.023	0.187
	morphotype × period	1	0.046	0.046	0.730	0.009	0.399
	residuals	37	2.315	0.063	NA	0.466	NA
Standard length	morphotype	1	2.701	2.701	39.112	0.495	0.000
	period	1	0.147	0.147	2.128	0.027	0.153
	morphotype × period	1	0.052	0.052	0.747	0.010	0.393
	residuals	37	2.555	0.069	NA	0.468	NA
Body weight	morphotype	1	26.762	26.762	40.397	0.508	0.000
	period	1	1.021	1.021	1.540	0.019	0.222
	morphotype × period	1	0.407	0.407	0.614	0.008	0.438
	residuals	37	24.512	0.663	NA	0.465	NA

**Note:** The morphotype of *P. hermanni*, which refers to the snout form, was excluded from this factorial analysis because only the short-snouted form was available; η² is reported as an effect size for each model term.

At the same time, explicit comparisons of variance indicated that the most pronounced temporal morphological signal was not a change in mean size, but rather a reduction in size variation within the wide-snouted *P. kaufmanni* (Table S4). For this morphotype, the variance in TL, FL, and SL was significantly higher in 1996 compared to the period from 2019 to 2024, as demonstrated by both F-tests (*p* = 0.007, 0.002, and 0.002, respectively) and Levene’s tests (*p* = 0.012, 0.017, and 0.003, respectively). Although body weight showed a similar trend in direction, the level of statistical support varied across the methods used (F-test, *p* = 0.110; Levene’s test, *p* = 0.017). By contrast, no significant temporal variance differences were detected in narrow-snouted *P. kaufmanni* or short-snouted *P. hermanni* (Table S4). Together, these results indicate that the clearest morphological change through time is a reduction in body-size dispersion, especially in the wide-snouted form, rather than a significant shift in mean size.

### Temporal decline in mitochondrial genetic diversity

We tracked changes in mitochondrial genetic diversity over time by comparing historical (1997–2005) *cyt b* and CR sequences deposited in NCBI, and current sequences from 31 individuals collected between 2020 and 2024 (Fig. 2D, E, Table 2, and Table S5). Descriptive comparisons showed substantial temporal declines in mitochondrial diversity across both *P. kaufmanni* morphotypes. For *cyt b*, nucleotide diversity (π) in wide-snouted individuals declined 6.98-fold from 0.00614 ± 0.00307 to 0.00088 ± 0.00044, accompanied by an 85.7% reduction in segregating sites (from 7 to 1). The narrow-snouted morphotype exhibited a similar pattern, with π decreasing from 0.00263 ± 0.00132 to 0.00088 ± 0.00044. When *cyt b* sequences from both morphotypes were pooled, π declined 9.86-fold, from 0.00424 ± 0.00122 to 0.00043 ± 0.00012, despite a substantial increase in sampling effort (from 4 to 26 sequences), and the descriptive indices also declined (segregating sites from 9 to 3; haplotypes from 4 to 4; Table S5), consistent with widespread loss of mitochondrial diversity across a broader population range. Although CR sequences showed higher baseline diversity, they followed a comparable trajectory of contraction. In the wide-snouted morphotype, π decreased 28.05-fold (from 0.04011 ± 0.02006 to 0.00143 ± 0.00071), while the narrow-snouted form experienced a 2.80-fold decrease (from 0.04052 ± 0.02026 to 0.01447 ± 0.00723). When CR sequences were pooled, π declined 5.74-fold from 0.04471 ± 0.011 to 0.00779 ± 0.00124, and segregating sites decreased by 53.3% (from 45 to 21). In *P. hermanni*, genetic erosion was also pronounced (Fig. 2E and Table 2). Within the short-snouted morphotype, *cyt b* diversity declined 2.99-fold, from 0.00527 ± 0.00263 to 0.00176 ± 0.00088. Pooled data from both morphotypes revealed a 3.34-fold reduction in π (from 0.00468 ± 0.00135 to 0.0014 ± 0.00042). CR sequences exhibited similar trends, with π in short-snouted specimens decreasing 1.81-fold (from 0.02699 ± 0.01349 to 0.01493 ± 0.00746), and pooled samples showing a 2.02-fold reduction (from 0.01771 ± 0.00503 to 0.00875 ± 0.00218).

**Table 2:** Temporal comparisons of haplotype diversity and nucleotide diversity in *Pseudoscaphirhynchus kaufmanni* and *P. hermanni* based on *cyt b* and control region sequences

Species	Gene	Morphotype	Sample size (N)		Haplotype diversity (Hd)				Nucleotide diversity (π)				
					Mean ± SE		Z-score	*P*-value	Mean ± SE		Z-score	*P*-value	π % decline
			1997–2005	2020–2024	1997–2005	2020–2024			1997–2005	2020–2024			
*P. kaufmanni*	*cyt b*	Wide-snouted	2	2	1.0000 ± 0.35355	1.0000 ± 0.35355	0.000	1.000	0.0061 ± 0.00217	0.0009 ± 0.00031	2.399	0.017	85.67
		Narrow-snouted	2	2	1.0000 ± 0.35355	1.0000 ± 0.35355	0.000	1.000	0.0026 ± 0.00093	0.0009 ± 0.00031	1.779	0.075	66.54
		Pooled (matched)	4	4	1.0000 ± 0.08850	0.8330 ± 0.11100	1.176	0.239	0.0042 ± 0.00061	0.0009 ± 0.00015	5.349	0.000	79.25
		Pooled (all samples)	4	26	1.0000 ± 0.08850	0.4520 ± 0.02138	6.019	0.000	0.0042 ± 0.00061	0.0004 ± 0.00002	6.241	0.000	89.86
	CR	Wide-snouted	2	2	1.0000 ± 0.35355	1.0000 ± 0.35355	0.000	1.000	0.0401 ± 0.01418	0.0014 ± 0.00050	2.725	0.006	96.43
		Narrow-snouted	2	2	1.0000 ± 0.35355	1.0000 ± 0.35426	0.000	1.000	0.0405 ± 0.01433	0.0145 ± 0.00511	1.713	0.087	64.29
		Pooled (matched)	4	4	1.0000 ± 0.08850	0.8330 ± 0.11100	1.176	0.239	0.0350 ± 0.00405	0.0107 ± 0.00160	5.576	0.000	69.33
		Pooled (all samples)	4	26	1.0000 ± 0.08850	0.7720 ± 0.01530	2.539	0.011	0.0447 ± 0.00550	0.0078 ± 0.00024	6.706	0.000	82.58
*P. hermanni*	*cyt b*	Short-snouted	2	2	1.0000 ± 0.35355	1.0000 ± 0.35355	0.000	1.000	0.0053 ± 0.00186	0.0018 ± 0.00062	1.790	0.074	66.60
		Pooled (all samples)	3	5	1.0000 ± 0.15704	0.9000 ± 0.07200	0.579	0.563	0.0047 ± 0.00078	0.0014 ± 0.00019	4.091	0.000	70.09
	CR	Short-snouted	2	2	1.0000 ± 0.35355	1.0000 ± 0.35355	0.000	1.000	0.02700 ± 0.00954	0.0149 ± 0.0053	1.106	0.269	44.68
		Pooled (all samples)	3	5	1.0000 ± 0.15704	1.0000 ± 0.05635	0.000	1.000	0.0177 ± 0.00290	0.0088 ± 0.00097	2.925	0.003	50.59

Comparisons involving only two sequences per period are shown for completeness but should be interpreted cautiously.

Hd and π were formally compared between historical (1997–2005) and recent (2020–2024) samples using two-tailed Z-tests based on the reported standard errors (SE); π % decline was calculated as ((π(1997–2005)-π(2020–2024))/π(1997–2005)*100).

Pooled (matched): wide- and narrow-snouted samples of *P. kaufmanni* pooled at a matched sampling level in each period.

Pooled (all samples): wide- and narrow-snouted samples pooled using all available sequences in each period for *P. kaufmanni*; and short-snouted samples and long-snouted records from a previous study for *P. hermanni*.

To formalize these temporal comparisons, we compared haplotype diversity (Hd) and π between historical and recent samples using two-tailed Z-tests based on the reported standard errors (Table 2). These analyses showed that the clearest and most consistent signal was a decline in π, particularly in pooled comparisons of *P. kaufmanni*. For *cyt b*, π declined significantly in wide-snouted *P. kaufmanni* (Z = 2.399, *p* = 0.017), whereas the decrease in narrow-snouted individuals was slighter (Z = 1.779, *p* = 0.075); both comparisons, however, were based on only two sequences per period and should therefore be interpreted cautiously. In pooled comparisons of *P. kaufmanni*, the temporal reduction in π was strongly supported for both *cyt b* (matched pooled: Z = 5.349, *p* < 0.001; all samples pooled: Z = 6.241, *p* < 0.001) and CR (matched pooled: Z = 5.576, *p* < 0.001; all samples pooled: Z = 6.706, *p* < 0.001). Hd showed the same general tendency in some comparisons, but statistical support was less consistent: it remained unchanged in the smallest subgroups, was non-significant in matched pooled comparisons, and declined significantly only in the pooled all-samples comparisons for *cyt b* (Z = 6.019, *p* < 0.001) and CR (Z = 2.539, *p* = 0.011) in *P. kaufmanni* (Table 2).

In *P. hermanni*, formal comparisons also revealed time-related decreases in π; however, the evidence was more robust when analyzing combined data rather than smaller subgroups. For *cyt b*, the decline in short-snouted specimens did not reach conventional significance (Z = 1.790, *p* = 0.074), whereas pooled samples showed a significant reduction (Z = 4.091, *p* < 0.001). For CR, the short-snouted comparison was likewise non-significant (Z = 1.106, *p* = 0.269), but the pooled comparison remained significant (Z = 2.925, *p* = 0.003). Hd did not differ significantly through time in *P. hermanni* (Table 2). Overall, the descriptive pattern of mitochondrial erosion over time was generally supported by the formal analyses, with π and pooled comparisons providing the strongest evidence due to their reduced reliance on extremely small subgroup sample sizes.

### Poaching driven by fertility beliefs is a primary driver of *P. kaufmanni* population collapse

**Fig. 3:**
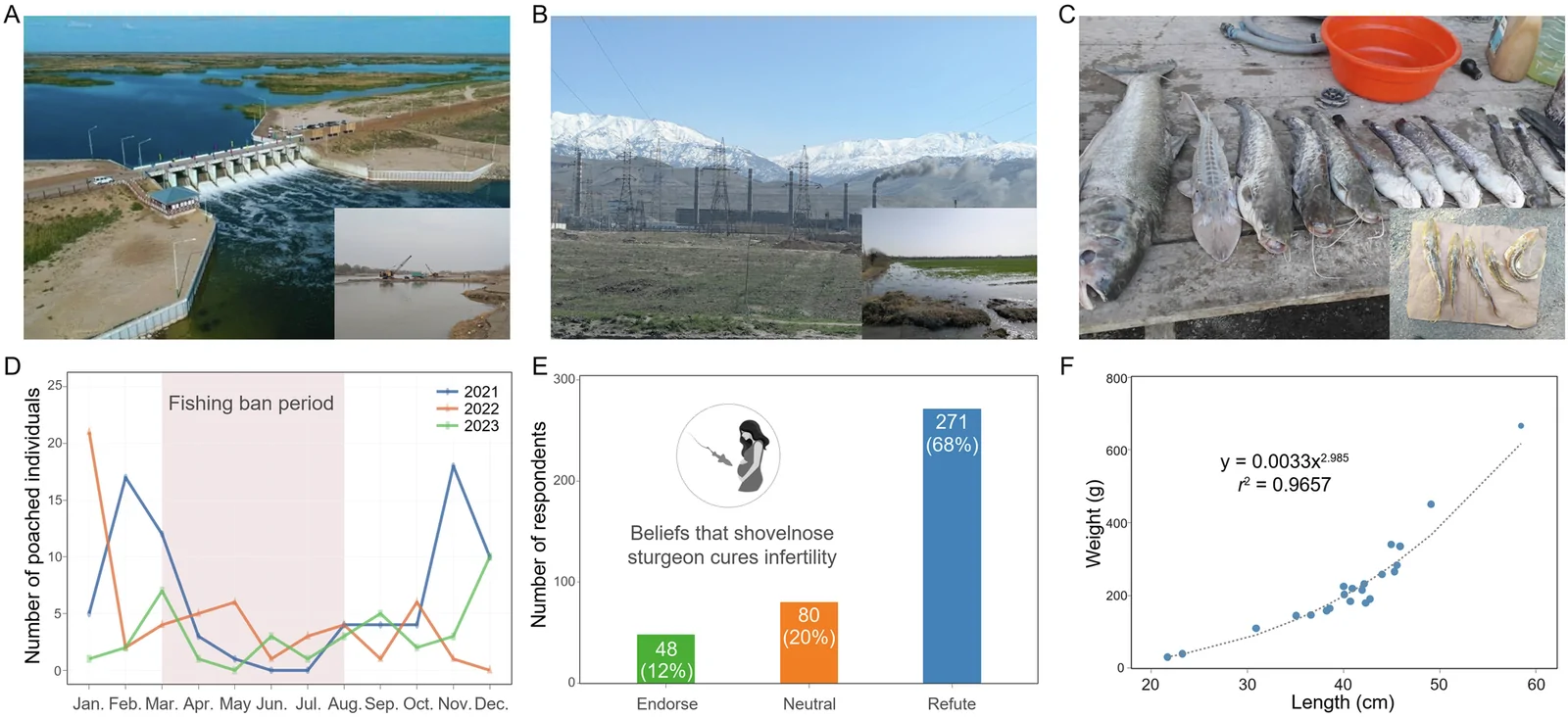
Threats contributing to population decline in Amu Darya false shovelnose sturgeons, with poaching and illegal trade intensified by fertility-related beliefs. **A–C** Habitat degradation from damming and gravel excavation, industrial and agricultural pollution, and poaching and illegal trade. **D** Monthly counts of illegally traded *P. kaufmanni* from 2021 to 2023. **E** Surveyed beliefs in the purported fertility benefits of *P. kaufmanni* in Khorezm, Uzbekistan. **F** Length-weight relationship of *P. kaufmanni* in the lower Amu Darya River.

Although Amu Darya false shovelnose sturgeons face multiple threats (e.g., river fragmentation, damming, industrial discharge, and agrochemical contamination) (Fig. 3A, B), they have also become a focal target of sustained illegal exploitation (Fig. 3C). However, the full extent of poaching remains underreported. Our monthly monitoring conducted in the Khorezm region between 2021 and 2023 documented 170 illegally harvested individuals sold in local fish markets (Fig. 3D). Poaching intensity peaked in autumn and winter, particularly between November and February, with the highest recorded takes occurring in November 2021 (18 individuals) and January 2022 (21 individuals) (Fig. 3D). The latter event took place within a 10 km stretch of the river near Khanka. Although a legal fishing ban is in effect from March to August, illegal harvest still occurred at lower levels (Fig. 3D). Notably, seizures during the spawning season included 23 individuals in March and 9 in April across the study period (Fig. 3D), indicating that breeding cohorts remain affected.

The principal driver of this targeted exploitation is market demand underpinned by persistent ethnozoological beliefs in the medicinal and aphrodisiac properties of the species, particularly for infertility treatment (Fig. 3E). This belief, rooted in pre-Islamic Khorezm mythology, describes false shovelnose sturgeons as having ingested the seed of Adam and thus acquiring fertility-enhancing powers. It remains deeply embedded in cultural consciousness (Nuraddyn aka, pers. comm.). Our social surveys showed that ~12% of 386 respondents from the Khorezm region endorsed this belief (Fig. 3E). This proportion is likely an underestimate because our sample is predominantly digitally connected and formally educated, potentially underrepresenting traditional knowledge holders. As wild stocks have declined and fish have become smaller on average, demand has shifted toward the largest individuals, providing fishers with a clear incentive to target big morphotypes for higher profits. In Khorezm and Karakalpakstan, the wide-snouted *P. kaufmanni* weighing more than 300 g can sell for as much as USD 200 per fish. That price premium encourages riskier, higher-payoff illegal fishing and further increases pressure on already depleted populations. Notably, this demand is not confined to the general public; anecdotal reports suggest that some local government personnel also take part in—and endorse—these practices (pers. comm., anonymous).

The length-weight relationship (LWR) in *P. kaufmanni* supports poaching as the most likely driver of population collapse. The LWR was well described by the model W = 0.0033 × L^2.985^ (R^2^ = 0.9657; Fig. 3F), indicating a strong and consistent growth relationship. The resulting parameters supported near-isometric growth, with an intercept of *a* = 0.0033 (95% CI: 0.0012–0.0086) and a slope of *b* = 2.985 (95% CI: 2.723–3.247). Notably, the confidence interval for *b* encompassed the cubic value of 3 (*p* > 0.05). These parameters indicate proportional increases in length and weight throughout development. This growth pattern aligns well with the ecophysiological theory, which predicts isometry under stable food supply, where energy allocation between structural growth and metabolic maintenance remains balanced^36,37^. As we do not observe a clear allometric shift that would typically signal strong growth constraints due to habitat deterioration, the simplest interpretation is that population losses are primarily driven by human harvesting. This inference aligns with field observations of intensified poaching and CITES seizure records across the species’ native range.

## Discussion

Amu Darya false shovelnose sturgeons appear to be undergoing a multi-driver collapse, characterized by range contraction, phenotypic degradation, and loss of genetic diversity—signs that echo the declines of sturgeon populations worldwide^38^. Catastrophic habitat loss has been primarily driven by anthropogenic alterations to hydrological systems. Large water-control and hydropower projects, particularly the Tuyamuyun and Takhiatash dams constructed to sustain cotton irrigation, have fundamentally altered fluvial dynamics. The reduced discharge velocity and increased water transparency have significantly disrupted the sediment-dependent spawning ecophysiology^13^. Additionally, downstream sections receive agrochemical inputs from agricultural activities^39^. The river is also physically broken up. Canals, such as Kyzketken, Shovot, Amu-Bukhara, and Amu-Zang, together with pumping stations, effectively divide the system into isolated fragments, leaving small groups of fish both disconnected and vulnerable to exploitation. Although some local water-quality indicators have improved since 2000, persistent regulated flows and weak monitoring of harvest in irrigation channels have hindered populations’ recovery^16^. In the end, lost connectivity, disrupted sediment transport, and ineffective fisheries management have significantly reduced the available habitat range.

Morphological data revealed morphotype-specific responses that are consistent with human-driven selection. For wide-snouted *P. kaufmanni*, individuals have undergone pronounced phenotypic homogenization, characterized by reduced body-size variance and significant body-weight loss. This type of phenotypic homogenization is consistent with selective removal of larger individuals through targeted poaching^40,41^. The disproportionate loss of large, highly fecund individuals may further reduce fecundity and reproductive success^42^. While the narrow-snouted morphotype tells a different story. Despite a severe decline in population size, the remaining fish appear larger, which fits a density-dependent growth release scenario: fewer competitors can mean more food per fish^43^. However, for a small, collapsing population, genetic drift can also leave a strong fingerprint. If growth-associated alleles happen to rise in frequency and become fixed, that could shift body size upward, but at a cost: the same demographic conditions that amplify drift also increase inbreeding risk and can undermine long-term persistence^44^. At the same time, because historical sample sizes for some groups, especially *P. hermanni*, remain very small, any interpretation of morphotype-specific temporal differences beyond the wide-snouted *P. kaufmanni* signal should remain cautious.

Beyond these phenotypic changes, genomic evidence further indicated that the evolutionary potential continues to erode. Mitogenomic analyses revealed a 9.86-fold decline in *cyt b* diversity and a 5.74-fold loss in CR diversity in *P. kaufmanni* since the 1990s, signaling a severe contraction in standing genetic variation. The concurrent collapse in segregating sites suggests disintegration of historical population substructure, likely exacerbated by fragmentation-induced isolation^45,46^. Similarly, the 3.34-fold reduction in *cyt b* diversity in *P. hermanni* indicates critical erosion of standing variation and diminished resilience to emerging stressors. Given the near-total absence of juveniles, this erosion of mitochondrial diversity increases the risk of mutational accumulation, particularly under conditions of persistent recruitment failure in fragmented freshwater systems^47,48^. The lack of fin-ray age data in this study limits direct inference on recruitment dynamics and age structure. Future research should prioritize the collection of complete, well-preserved fin rays to enable robust age estimation and clarify whether the reduction in juveniles reflects chronic recruitment failure, age truncation, or both. Our Z-test comparisons of Hd and π further emphasize this uncertainty. In particular, declines in π showed the most consistent statistical support, especially in pooled comparisons of *P. kaufmanni*, whereas support for Hd was less consistent across markers and morphotypes. This difference is important because it shows that the signal of genetic erosion is strongest at the level of sequence divergence, while some of the smallest subgroup comparisons are still limited by small sample sizes. Even so, decreases in S and H and the significant declines in π both strongly support an ongoing loss of mitochondrial variation over time.

Among the dominant drivers of widespread false shovelnose sturgeon degeneration, river damming remains a globally prevalent threat to migratory Acipenseriformes^49,50^. However, in the case of the false shovelnose sturgeons, illegal poaching and trade persist as a pervasive and critical extinction threat to remnant populations^51^, intensified by culturally entrenched ethnozoological beliefs in the medicinal properties of *P. kaufmanni*. To advance understanding of the sociocultural drivers behind illegal harvest, future work should prioritize in-person surveys in rural communities with limited digital access, where traditional ethnozoological beliefs are likely common but often overlooked. The poaching we documented, involving 170 *P. kaufmanni* individuals taken between 2021 and 2023 (Fig. 3D), reflects extraction levels comparable to those observed in the Danube basin, where genetic and isotope studies suggest that 21% of market fish originated from illegal wild captures^52^. Although this figure underscores the severity of illegal harvest pressure, quantifying its demographic consequences without robust population estimates remains challenging. This limitation underscores an urgent need for quantitative population modeling to evaluate extinction thresholds under continued exploitation. Meanwhile, enforcement remains a major weak link. Along much of the Amu Darya—especially across the Afghanistan–Uzbekistan–Turkmenistan corridor—governance is fragmented or absent, and the irrigation network still gives poachers easy access to habitats that are already fragmented and isolated. Without immediate and coordinated action, the ongoing illegal harvest may drive these sturgeons to extinction, with stochastic disturbances, such as droughts or disease outbreaks further destabilizing populations already reduced to critically small effective sizes^53^.

The current IUCN Red List assessment largely relies on population size and geographic range, but for Amu Darya false shovelnose sturgeons, that lens captures only part of the collapse. In our case, the decline is also accompanied by significant habitat loss, body miniaturization, and reduced genetic diversity. Our revised analyses suggest that this collapse should be understood not only as a numerical and spatial decline, but also as a loss of phenotypic and genetic variability. In particular, the contraction of body-size variance in wide-snouted *P. kaufmanni* and the formal reduction in mitochondrial diversity, especially π, indicate that erosion is occurring at multiple biological levels simultaneously. Given the severity of the decline, five immediate and coordinated conservation actions are necessary: (1) strengthened nationwide enforcement to stop illegal harvest; (2) managing dam discharges to recover the sediment transport and flow conditions required for spawning; (3) targeted outreach to counter long-held beliefs about the medicinal value of the big Amu Darya false shovelnose sturgeon; (4) developing and enlarging hatchery programs to produce juveniles for restocking; and (5) adding biomimetic fish passages to low-head barriers to recover longitudinal connectivity. Implementing these interventions will require substantial investment in local conservation capacity, backed by political commitment to tackle longstanding governance and resource constraints.

Besides these species-level conservation actions, basin-level planning is equally important. Such planning could draw on lessons from successful riverine recovery initiatives, as those implemented in the Yangtze River^49^. These measures can slow the slide in the short term, but long-term recovery will depend on the eco-evolutionary processes that determine how extinction risk actually unfolds. Thus, future conservation efforts should incorporate high-quality species genomes and whole-genome resequencing data. These datasets will enable us to dissect the drivers currently acting in concert—harvest selection that shifts age-specific variation, genome erosion that erodes adaptive genetic diversity, and cultural demand that reinforces non-adaptive pressure—and then use that understanding to evaluate genomic-rescue strategies for these ancient, Mesozoic-origin evolutionary lineages.

## Supplementary information

Supplementary Tables

## Data Availability

All raw whole-genome resequencing data (GSA: CRA024581) in this study were deposited in the Genome Sequence Archive of the National Genomics Data Center, China National Center for Bioinformation/Beijing Institute of Genomics, Chinese Academy of Sciences, and are publicly accessible at https://ngdc.cncb.ac.cn/gsa.
