# Supplementary material for: Multidimensional crisis in the conservation of Amu Darya false shovelnose sturgeons
Source: NPJ Biodivers. 2026 Jun 1;5:32. doi: 10.1038/s44185-026-00141-w (PMC13627660; doi:10.1038/s44185-026-00141-w)
Supplement: Supplementary file 1 [file 44185_2026_141_MOESM1_ESM.pdf]

Table S1. Recordings and samples used in the genetic diversity estimation of the Amu Darya shovelnose sturgeons

No.	Species	Sample ID	Morphotype	Place	Date	Gene/Genome	Accession number	Sample used in purpose	Latitude	Longitude	Evidence	Reference
1	*P. kaufmanni*	Wide–1	Wide–snouted	Kerki, Turkmenistan	1997–2005	CR/cyt *b*	EF484347/EF484340	Genetic diverstiy	NA	NA	Bycatch	Dillman *et al* ., 2007
2	*P. kaufmanni*	Wide–2	Wide–snouted	Kerki, Turkmenistan	1997–2005	CR/cyt *b*	EF484350/EF484343	Genetic diverstiy	NA	NA	Bycatch	Dillman *et al* ., 2007
3	*P. kaufmanni*	Narrow–1	Narrow–snouted	Kerki, Turkmenistan	1997–2005	CR/cyt *b*	EF484346/EF484339	Genetic diverstiy	NA	NA	Bycatch	Dillman *et al* ., 2007
4	*P. kaufmanni*	Narrow–2	Narrow–snouted	Kerki, Turkmenistan	1997–2005	CR/cyt *b*	EF484351/EF484344	Genetic diverstiy	NA	NA	Bycatch	Dillman *et al* ., 2007
5	*P. kaufmanni*	Pkau–C1	Wide–snouted	Beruni, Uzbekistan	20/5/2020	WGS	PRJCA036986	Genetic diverstiy/morphology	41.6688	60.6854	Market	This study
6	*P. kaufmanni*	Pkau–C2	Wide–snouted	Urgench, Uzbekistan	21/5/2020	WGS	PRJCA036986	Genetic diverstiy/morphology	41.6608	60.6893	Market	This study
7	*P. kaufmanni*	Pkau–C3	Wide–snouted	Pitnak, Uzbekistan	27/1/2021	WGS	PRJCA036986	Genetic diverstiy/morphology	41.2444	61.3034	Market	This study
8	*P. kaufmanni*	Pkau–C4	Wide–snouted	Ayakchi, Uzbekistan	2021/1/2	WGS	PRJCA036986	Genetic diverstiy/morphology	42.1614	60.1875	Market	This study
9	*P. kaufmanni*	Pkau–C5	Wide–snouted	Ayakchi, Uzbekistan	2021/1/2	WGS	PRJCA036986	Genetic diverstiy/morphology	42.1614	60.1875	Market	This study
10	*P. kaufmanni*	Pkau–C6	Wide–snouted	Miskin, Uzbekistan	2021/4/2	WGS	PRJCA036986	Genetic diverstiy/morphology	41.3862	61.1610	Market	This study
11	*P. kaufmanni*	Pkau–C7	Wide–snouted	Miskin, Uzbekistan	2021/4/2	WGS	PRJCA036986	Genetic diverstiy/morphology	41.3782	61.1692	Market	This study
12	*P. kaufmanni*	Pkau–C8	Wide–snouted	Xazorasp, Uzbekistan	2021/4/2	WGS	PRJCA036986	Genetic diverstiy/morphology	41.4103	61.0936	Market	This study
13	*P. kaufmanni*	Pkau–C9	Wide–snouted	Tortkol, Uzbekistan	18/10/2021	WGS	PRJCA036986	Genetic diverstiy/morphology	41.4401	61.0354	Market	This study
14	*P. kaufmanni*	Pkau–C10	Wide–snouted	Xazorasp, Uzbekistan	23/11/2021	WGS	PRJCA036986	Genetic diverstiy/morphology	41.4185	61.0725	Market	This study
15	*P. kaufmanni*	Pkau–C11	Wide–snouted	Xonqa, Uzbekistan	2021/4/12	WGS	PRJCA036986	Genetic diverstiy/morphology	41.5861	60.8305	Market	This study
16	*P. kaufmanni*	Pkau–C12	Wide–snouted	Beruni, Uzbekistan	20/12/2021	WGS	PRJCA036986	Genetic diverstiy/morphology	41.5890	60.8469	Market	This study
17	*P. kaufmanni*	Pkau–C13	Wide–snouted	Xonqa, Uzbekistan	20/12/2021	WGS	PRJCA036986	Genetic diverstiy/morphology	41.5994	60.8203	Market	This study
18	*P. kaufmanni*	Pkau–C14	Wide–snouted	Beruni, Uzbekistan	20/12/2021	WGS	PRJCA036986	Genetic diverstiy/morphology	41.6106	60.8131	Market	This study
19	*P. kaufmanni*	Pkau–C15	Wide–snouted	Urgench, Uzbekistan	20/1/2022	WGS	PRJCA036986	Genetic diverstiy/morphology	41.6291	60.7631	Market	This study
20	*P. kaufmanni*	Pkau–C16	Wide–snouted	Gurlan, Uzbekistan	14/2/2022	WGS	PRJCA036986	Genetic diverstiy/morphology	41.9647	60.4031	Market	This study
21	*P. kaufmanni*	Pkau–C17	Wide–snouted	Kipchak, Uzbekistan	19/3/2022	WGS	PRJCA036986	Genetic diverstiy	42.2659	60.0077	Market	This study
22	*P. kaufmanni*	Pkau–C18	Wide–snouted	Xazorasp, Uzbekistan	2022/11/7	WGS	PRJCA036986	Genetic diverstiy	41.4209	61.0653	Market	This study
23	*P. kaufmanni*	Pkau–C19	Wide–snouted	Xonqa, Uzbekistan	29/8/2022	WGS	PRJCA036986	Genetic diverstiy	41.5389	60.8217	Market	This study
24	*P. kaufmanni*	Pkau–C20	Wide–snouted	Xazorasp, Uzbekistan	2022/12/10	WGS	PRJCA036986	Genetic diverstiy	41.3271	61.2505	Market	This study
25	*P. kaufmanni*	Pkau–C21	Wide–snouted	Tortkol, Uzbekistan	2022/11/12	WGS	PRJCA036986	Genetic diverstiy	41.5195	60.9512	Market	This study
26	*P. kaufmanni*	Pkau–C22	Wide–snouted	Urgench, Uzbekistan	30/8/2023	WGS	PRJCA036986	Genetic diverstiy	41.6356	60.7386	Market	This study
27	*P. kaufmanni*	Pkau–C23	Wide–snouted	Beruni, Uzbekistan	29/9/2023	WGS	PRJCA036986	Genetic diverstiy	41.6985	60.6708	Market	This study
28	*P. kaufmanni*	Pkau–C24	Wide–snouted	Yangibazar, Uzbekistan	29/12/2023	WGS	PRJCA036986	Genetic diverstiy	41.7741	60.5861	Market	This study
29	*P. kaufmanni*	Pkau–D1	Narrow–snouted	Xazorasp, Uzbekistan	2019/8/8	NA	NA	Morphology	41.3450	61.2336	Market	This study
30	*P. kaufmanni*	Pkau–D2	Narrow–snouted	Urgench, Uzbekistan	18/10/2023	WGS	PRJCA036986	Genetic diverstiy/morphology	41.6027	60.7871	Market	This study
31	*P. kaufmanni*	Pkau–D3	Narrow–snouted	Xazorasp, Uzbekistan	2024/5/2	WGS	PRJCA036986	Genetic diverstiy/morphology	41.4116	61.0846	Market	This study
32	*P. kaufmanni*	Pkau–D4	Narrow–snouted	Xonqa, Uzbekistan	2024/11/4	NA	NA	Captive breeding dead	41.4294	61.0422	By–catch	This study
33	*P. kaufmanni*	NA	Wide–snouted	Kushkupir, Uzbekistan	15/3/2024	NA	NA	Recaptured	41.5457	60.1413	Bycatch recaptured	This study
34	*P. kaufmanni*	NA	Wide–snouted	Nukus Balyk, Uzbekistan	2024/4/4	NA	NA	Captive breeding	41.4120	60.8440	Bycatch captive breeding	GBIF
35	*P. kaufmanni*	NA	Wide–snouted	Nukus Balyk, Uzbekistan	2024/7/10	NA	NA	Captive breeding	41.3195	61.2399	Bycatch captive breeding	GBIF
36	*P. kaufmanni*	NA	Narrow–snouted	Nukus Balyk, Uzbekistan	16/11/2024	NA	NA	Captive breeding	41.5694	61.1925	Bycatch captive breeding	GBIF
37	*P. kaufmanni*	NA	Narrow–snouted	Nukus Balyk, Uzbekistan	23/11/2024	NA	NA	Captive breeding	41.5273	61.1751	Bycatch captive breeding	GBIF
38	*P. kaufmanni*	NA	Narrow–snouted	Nukus Balyk, Uzbekistan	2024/12/12	NA	NA	Captive breeding	41.4014	60.9619	Bycatch captive breeding	GBIF
39	*P. kaufmanni*	NA	Wide–snouted	Kyzylravat, Uzbekistan	17/4/2005	NA	NA	NA	40.5428	62.0745	Bycatch	GBIF
40	*P. kaufmanni*	NA	Narrow–snouted	Khatlon, Tajikistan	14/4/2012	NA	NA	NA	37.3664	68.4820	Bycatch	GBIF
1	*P. hermanni*	Short–1	Short–snouted	Kerki, Turkmenistan	1997–2005	CR/cyt *b*	EF484345/EF484338	Genetic diversity	NA	NA	Bycatch	Dillman *et al* ., 1997
2	*P. hermanni*	Short–2	Short–snouted	Kerki, Turkmenistan	1997–2005	CR/cyt *b*	EF484349/EF484342	Genetic diversity	NA	NA	Bycatch	Dillman *et al* ., 1997
3	*P. hermanni*	Long–2	Long–snouted	Kerki, Turkmenistan	1997–2005	CR/cyt *b*	EF484348/EF484341	Genetic diversity	NA	NA	Bycatch	Dillman *et al* ., 1997
4	*P. hermanni*	Pher–D1	Short–snouted	Xonqa, Uzbekistan	19/1/2020	WGS	PRJCA036986	Genetic diversity/morphology	41.4896	60.9558	Bycatch	This study
5	*P. hermanni*	Pher–D2	Short–snouted	Xonqa, Uzbekistan	21/1/2020	WGS	PRJCA036986	Genetic diversity/morphology	41.4896	60.9558	Bycatch	This study
6	*P. hermanni*	Pher–D3	Short–snouted	Xazorasp, Uzbekistan	25/1/2020	WGS	PRJCA036986	Genetic diversity/morphology	41.4183	61.0812	Bycatch	This study
7	*P. hermanni*	Pher–D4	Short–snouted	Pitnak, Uzbekistan	28/3/2020	WGS	PRJCA036986	Genetic diversity/morphology	41.2397	61.3151	Bycatch	This study
8	*P. hermanni*	Pher–D5	Short–snouted	Urgench, Uzbekistan	2020/3/4	WGS	PRJCA036986	Genetic diversity/morphology	41.6338	60.7566	Bycatch	This study
9	*P. hermanni*	NA	Short–snouted	Urgench, Uzbekistan	29/3/2021	NA	NA	Morphology	41.6060	60.7854	Bycatch	This study
10	*P. hermanni*	NA	Short–snouted	Petroaleksandrovsk, Uzbek	2021/10/11	NA	NA	Recaptured	41.4768	60.9602	Bycatch recaptured	This study
11	*P. hermanni*	NA	Short–snouted	Xazorasp, Uzbekistan	15/12/2021	NA	NA	Recaptured	41.4115	61.0850	Bycatch recaptured	This study
12	*P. hermanni*	NA	Short–snouted	Sarybay, Uzbekistan	2022/5/4	NA	NA	Recaptured	41.5300	60.9293	Bycatch recaptured	This study
13	*P. hermanni*	NA	Short–snouted	Nukus, Uzbekistan	14/5/2022	NA	NA	Recaptured	42.5337	59.5903	Bycatch recaptured	This study
14	*P. hermanni*	NA	Short–snouted	Urgench, Uzbekistan	24/6/2023	NA	NA	Recaptured	41.5437	60.6041	Bycatch recaptured	This study
15	*P. hermanni*	NA	Short–snouted	Yangibazar, Uzbekistan	26/11/2023	NA	NA	Recaptured	41.7306	60.6422	Bycatch recaptured	This study
16	*P. hermanni*	NA	Short–snouted	Tortkol, Uzbekistan	2024/8/11	NA	NA	NA	41.5835	61.1631	Bycatch	GBIF

Note: This list does not include detaled information of all 170 market individuals.

Table S2. Summary statistics of log-transformed morphometric traits and results of the Shapiro-Wilk normality test

Species	Morphotype	Period	Trait	N	Log-transformed values				Shapiro-Wilk test		
					Min	Max	Mean	SD	W	*P* -	Normality
P. kaufmanni	Wide-snouted	1996	Total length	16	2.9957	4.0164	3.6207	0.3428	0.8662	0.0238	No
			Fork length	16	2.9178	3.8959	3.5026	0.3363	0.8717	0.0289	No
			Standard length	16	2.7408	3.8067	3.3776	0.3546	0.8816	0.0410	No
			Weight	16	3.1781	6.4378	5.1667	1.0838	0.8799	0.0386	No
		2019–2024	Total length	16	3.4315	4.0681	3.7316	0.1318	0.9086	0.1106	Yes
			Fork length	16	3.3700	3.9154	3.5752	0.1142	0.8098	0.0037	No
			Standard length	16	3.2467	3.7979	3.4629	0.1227	0.9122	0.1263	Yes
			Weight	16	4.7075	6.4998	5.3853	0.4052	0.9208	0.1735	Yes
	Narrow-snouted	1996	Total length	6	2.4159	3.1224	2.9804	0.2783	0.5921	0.0004	No
			Fork length	6	2.3026	3.0204	2.8626	0.2779	0.6259	0.0009	No
			Standard length	6	2.1401	2.8507	2.7119	0.2839	0.5960	0.0004	No
			Weight	6	1.2528	3.5553	3.0832	0.9047	0.6067	0.0006	No
		2019–2024	Total length	3	3.0783	3.3006	3.1751	0.1139	0.9525	0.5804	Yes
			Fork length	3	2.9699	3.2408	3.1041	0.1355	0.9998	0.9704	Yes
			Standard length	3	2.8339	3.1171	2.9768	0.1416	0.9997	0.9693	Yes
			Weight	3	3.4544	4.2569	3.8060	0.4103	0.9561	0.5968	Yes
P. hermanni	Short-snouted	1996	Total length	2	3.0540	3.1355	3.0947	0.0576	NA	NA	Not tested (n < 3)
			Fork length	2	2.9704	3.0587	3.0146	0.0624	NA	NA	Not tested (n < 3)
			Standard length	2	2.8507	2.9339	2.8923	0.0588	NA	NA	Not tested (n < 3)
			Weight	2	3.2189	3.4965	3.3577	0.1963	NA	NA	Not tested (n < 3)
		2019–2024	Total length	6	2.7518	3.1183	3.0056	0.1379	0.8377	0.1248	Yes
			Fork length	6	2.6494	3.0043	2.8873	0.1357	0.8721	0.2345	Yes
			Standard length	6	2.5139	2.9131	2.7786	0.1445	0.8737	0.2413	Yes
			Weight	6	2.6769	3.5852	3.2475	0.3031	0.8254	0.0981	Yes

Table S3. Comparative morphometric features of *P. kaufmanni* and *P. hermanni* sampled from the lower and middle part of the Amu Darya River

Species	Morphotype	Period	Sampling site	Sampling size	Median			
					Total length (cm, without filament)	Fork length	Standard length	Weight (g)
P. kaufmanni	Wide-snouted	2019–	Lower Amu Darya	16	42.05	36.00	31.23	209.05
		1996	Middle Amu Darya	16	43.40	37.50	33.50	295.50
	Narrow-snouted	2019–	Lower Amu Darya	3	23.25	22.24	19.68	40.72
		1996	Middle Amu Darya	6	22.00	19.75	17.25	31.00
P. hermanni	Short-snouted	2019–	Lower Amu Darya	6	20.83	18.71	16.65	27.24
	Short-snouted	1996	Middle Amu Darya	2	22.10	20.40	18.05	29.00

Table S4. Variance tests (F-test and Levene's test) for morphometric traits by species and morphotype across two time periods

Species	Morphotype	Trait	Sample size (N)		F-test				Levene's test	
			1996	2019–2024	Variance (1996)	Variance (2019–2024)	F value	*p*	F value	*p*
P. kaufmanni	Wide-snouted	Total length	16	16	142.23	32.74	4.344	**0.007**	7.183	**0.012**
		Fork length	16	16	110.06	19.76	5.571	**0.002**	11.632	**0.002**
		Standard length	16	16	95.88	17.78	5.393	**0.002**	10.153	**0.003**
		Weight	16	16	37893.63	16178.69	2.342	0.110	6.397	**0.017**
	Narrow-snouted	Total length	6	3	20.12	7.77	2.591	0.603	0.045	0.838
		Fork length	6	3	16.08	9.22	1.744	0.807	0.001	0.974
		Standard length	6	3	12.32	7.76	1.589	0.859	0.001	0.980
		Weight	6	3	146.10	415.29	0.352	0.300	0.420	0.538
P. hermanni	Short-snouted	Total length	2	6	1.62	6.85	0.237	0.706	0.671	0.444
		Fork length	2	6	1.62	5.35	0.303	0.788	0.617	0.462
		Standard length	2	6	1.13	4.77	0.236	0.705	0.613	0.463
		Weight	2	6	32.00	48.28	0.663	0.905	0.005	0.946

Table S5. Temporal variation in the number of segregating sites (S) and haplotypes (H) in *Pseudoscaphirhynchus kaufmanni* and *P. hermanni* based on *cyt b* and control region sequences.

Species	Gene	Morphotype	Sample size (N)		Number of segregating sites (S)		Number of haplotypes (H)		Note
			1997–2005	2020–2024	1997–2005	2020–2024	1997–2005	2020–2024	
P. kaufmanni	cyt *b*	Wide	2	2	7	1	2	2	n = 2 per period; interpret cautiously
		Narrow	2	2	3	1	2	2	n = 2 per period; interpret cautiously
		Pooled (matched)	4	4	9	2	4	3	
		Pooled (all samples)	4	26	9	3	4	4	
	CR	Wide	2	2	28	1	2	2	n = 2 per period; interpret cautiously
		Narrow	2	2	28	10	2	2	n = 2 per period; interpret cautiously
		Pooled (matched)	4	4	41	13	4	3	
		Pooled (all samples)	4	26	45	21	4	10	
P. hermanni	cyt *b*	Short	2	2	6	2	2	2	n = 2 per period; interpret cautiously
		Pooled (all samples)	3	5	8	4	3	4	
	CR	Short	2	2	18	10	2	2	n = 2 per period; interpret cautiously
		Pooled (all samples)	3	5	17	12	3	5	

Notes:

S, number of segregating sites; H, number of haplotypes.

These indices are shown descriptively and were not subjected to the same formal testing framework as Hd and Pi because they are count-based summary measures strongly influenced by sample size.

Comparisons involving only two sequences per period should be interpreted cautiously due to limited sample size.Pooled (matched): wide- and narrow-snouted samples pooled at a matched sampling level in each period for *P. kaufmanni* .

Pooled (all samples): wide- and narrow-snouted samples pooled using all available sequences in each period for *P. kaufmanni* ; and short-snouted samples and long-snouted records from a previous study for *P. hermanni* .
